# Investigation into the Potential Migration of Nanoparticles from Laponite-Polymer Nanocomposites

**DOI:** 10.3390/nano8090723

**Published:** 2018-09-13

**Authors:** Johannes Bott, Roland Franz

**Affiliations:** Department of Product Safety and Analytics, Fraunhofer Institute for Process Engineering and Packaging (IVV), 85354 Freising, Germany; roland.franz@ivv.fraunhofer.de

**Keywords:** laponite, clay, nanomaterial, migration, diffusion, nanocomposites

## Abstract

In this study, the migration potential of laponite, a small synthetic nanoclay, from nanocomposites into foods was investigated. First, a laponite/ethylene vinyl acetate (EVA) masterbatch was compounded several times and then extruded into thin low-density polyethylene (LDPE) based films. This way, intercalation and partial exfoliation of the smallest type of clay was achieved. Migration of laponite was investigated using Asymmetric Flow Field-Flow Fractionation (AF4) with Multi-Angle Laser Light Scattering (MALLS) detection. A surfactant solution in which laponite dispersion remained stable during migration test conditions was used as alternative food simulant. Sample films with different loadings of laponite were stored for 10 days at 60 °C. No migration of laponite was found at a limit of detection of 22 µg laponite per Kg food. It can be concluded that laponite (representing the worst case for any larger structured type of clay) does not migrate into food once it is incorporated into a polymer matrix.

## 1. Introduction

Laponite is a synthetic colloidal layered silicate which is composed of disc-shaped crystals in the nanoscale size region. Laponite forms the same tetrahedral and octahedral 2:1 “sandwich” structure like most natural clays, e.g., montmorillonite [1]. In contrast, the primary structure (i.e., the disc-shaped crystals) is significant smaller than it is in naturally occurring clay minerals. Laponite crystals are of approximately 1 nm in thickness and have diameters of approximately 25 nm, only [2]. In general, clays are used as polymer additives nearly as long as polymers are on the market. From the beginning, clays are used as matrix fillers improving thermal and mechanical properties of the polymer significantly. In its exfoliated form, clay promises to enhance the barrier function plastic food contact materials (FCM) regarding oxygen or water vapor [3,4,5,6,7,8,9,10,11,12,13,14,15,16,17,18,19,20]. Laponites are mainly used as a rheology modifier or as film formers. They find applications in many consumer care products to improve suspension stability (e.g., cleaning products) and to adjust rheological behaviour (e.g., used as thickener in toothpaste). Laponites are also applied as a thin film on the surface of different materials like paper and polymeric films to produce food packaging that are antistatic or that have improved barrier properties regarding oxygen transmission [21]. Furthermore, the successful use of laponite disks in sustainable polymers like hydroxypropyl cellulose [22] and pectins [23] was demonstrated.

Due to its structural composition of ultrafine platelets with only 1 nm in thickness, laponite (and clay) has to be considered as a nanomaterial (NM) according to the definition of the European Union 2011/696/EU [24]. Nanomaterials in general promise many technical and economic benefits and are increasingly used. Therefore, an assessment of these materials from a safety perspective is always needed. This is, in particular, the case when such NMs are used as additives in FCM. It is generally accepted that a risk for the consumer is only given when exposure to NMs used as a polymer additive for food packaging is performed. In case of nanocomposites, exposure towards the NM would only be the case if the NM migrates out of the packaging into food. Within the last years the number of studies that investigated the migration potential of different types of NMs has increased and can be found summarized elsewhere [7,25,26]. In some case-examples practical evidence was already given that some NMs cannot migrate and theoretical considerations even showed that NMs used as additives in FCM are too large to migrate in general [26,27]. The use of NMs as additives in FCM made of plastics is regulated within the European Union in regulation EU/10/2011 [28] and its amendments [29,30,31]. Legal requirements demand that a risk assessment of NMs in FCM has to be performed on a case-by-case basis.

In this study, the migration potential of laponite from nanocomposites based on low-density polyethylene (LDPE) was investigated. Due to its small size compared to other nanoclays, laponite was selected as a model nanomaterial which will represent the worst-case in comparison with all other types of clay materials when considering migration being based on size-dependent Fickian diffusion [27].

## 2. Materials and Methods

### 2.1. Materials

Organically modified laponite RXG7308 (in the following: Laponite) (BYK Chemie GmbH, Moosburg, Germany) was provided both as a pure powder and also incorporated into ethylene-vinyl acetate (EVA) as a masterbatch (SO 9015, BYK Chemie GmbH, Moosburg, Germany) with a laponite content of 11%-m/m. The powder was used for analytical method development and the masterbatch was used to produce LDPE films with different contents of laponite for migration measurements.

The production of the LDPE films was performed in-house at Fraunhofer IVV. For this the SO 9015 masterbatch was first mixed with neat ethylene-vinyl acetate copolymer (Escorene Ultra FL 00226CC, 26% vinyl acetate, ExxonMobil Chemical Company, Houston, TX, USA) and compounded six times using a twin-screw compounder (Dr. Collin GmbH). With the preceded compounding strong shear forces are applied to the laponite stacks which shall provide better homogeneity in the LDPE films and partial intercalation/exfoliation of the layered laponite stacks. After compounding, the EVA/laponite masterbatch was mixed with LDPE (Lupolen 1806 H, LyondellBasell, Rotterdam, The Netherlands) and extruded to films with 2%, 4% and 6% laponite in the polymer using a flat film extruder (Dr. Collin GmbH). An LDPE film without laponite in the polymer was extruded as reference film.

### 2.2. Transmission Electron Microscopy (TEM)

TEM images of the polymeric film with the lowest laponite concentration were prepared by psi cube, Germany. With this technique the distribution and size characteristics of the laponite in the polymer was visualized. For sample preparation the polymeric film was subjected to cryo-ultra-thin-sectioning using a diamond knife.

### 2.3. Preparation of Laponite Reference Dispersions

Ultrapure water (TKA Genpure, Fisher Scientific GmbH, Schwerte, Germany) with 25000 mg/L of the surfactant Novachem (Postnova Analytics) and 200 mg/L of the biocide sodium azide (Merck Millipore, Darmstadt, Germany) was used as dispersant for a laponite stock dispersion. Dilutions of the initial stock dispersion were produced using ultrapure water with 2000 mg/L of the surfactant only. Ultrapure water with 200 mg/L sodium azide, without surfactants, was used as flowing liquid for the AF4. After preparation all solvents were filtered (0.1 µm Millipore filter disc).

For the stock dispersion 50.0 mg Laponite RXG7308 powder (BYK Chemie GmbH, Moosburg, Germany) was weighed out into a 50 mL polypropylene centrifuge vial and mixed with 20 mL of the dispersant. The centrifuge vial was then placed into an ice bath and the mixture was dispersed for 45 min using an ultra-sonication tip (Vibra Cell VC 50T, Sonics&Materials Inc., Newton, CT, USA, operated at 50 Watt, 20 kHz, 100% output), which was placed approximately 1 cm above the bottom of the vial. At the end of the dispersion experiment the dispersion was transferred quantitatively into a 200 mL perfluoroalkoxy alkane (PFA) measuring flask and filled up to the mark. This way a laponite dispersion with a nominal laponite concentration of 250 mg/L was prepared. The dispersion did neither showed sedimentation of laponite nor a strong milky turbidity, but was slightly opalescent. A complete exfoliation of the Laponite layers would result in clear dispersions, wherefore the slight opalescence was an indication that Laponite aggregates were broken into smaller units and at least intercalation of the Laponite stacks was successful, due to the high energy input of the ultra-sonication tip.

### 2.4. AF4 and MALLS Measurements

AF4 measurements were carried out with an “AF2000 MT Series mid temperature” (Postnova Analytics, Landsberg, Germany) to characterize, detect and quantify laponite particles in the ongoing migration experiments. The system was equipped with a 500 µm channel and a polyethersulfone membrane (cut-off: 10 kDa). For the determination of the particles size distribution a 21-angle-MALLS detector “PN3621” (Postnova Analytics, Landsberg, Germany) was used.

For diluted Laponite RXG7308 dispersions the AF4 conditions were optimised as follows: During the injection time of 15 min (this is tantamount to the focusing time), the cross flow is kept constant at 0.8 mL/min (start conditions). Within a transition time of 0.5 min the focusing of the sample is terminated and the elution of sample starts. The cross flow is kept constant for additional 5 min followed by a fast non-linear decline of the cross flow to 0 mL/min within 10.0 min, using a power gradient of 0.15. The channel is flushed by the detector flow for 35 min without any cross flow to deplete the channel completely. The detector flow is kept constant at 0.45 mL/min for the whole run. The channel was tempered to constant 40 °C. The method was used for sample injection volumes up to 2000 µL.

For laponite particles, a serial dilution from a 250 mg/L stock solution was made and standard solutions with 0 ng/mL (blank), 250 ng/mL, 500 ng/mL, 1000 ng/mL, 1500 ng/mL, 2000 ng/mL and 2500 ng/mL were measured. The detected signals of each standard dispersion were integrated at all detection angles (excluding the 7°, 12° and 164° angle) of the MALLS detector to obtain the peak area of the sample. The detected peak area is directly proportional to the injected mass.

Furthermore, the results of the light scattering experiment were used to calculate the sizes of dispersed laponite particles in form of the radius of gyration, *r_g_*.

### 2.5. Migration Test

For the migration studies simulants and test conditions were intended to be chosen according to Annex V of the European Plastics Regulation (EU) 10/2011 for long term storage at room temperature (more than 6 month) including hotfill (2 h at 70 °C or 15 min at 100 °C). Preliminary dispersion experiments showed that only in the 2000 mg/L Novachem surfactant solution sufficient dispersion stability of laponite particles could be achieved. Therefore, the surfactant solution (2000 mg/L Novachem and 200 mg/L sodium azide) was chosen as (alternative) food simulant.

Sample films were cut into squares to a defined area of 1 dm². The samples were stored in 30 mL polypropylene vials. Both samples and vials were blown out before with nitrogen to prevent contamination by dust, filled with 15 mL of the food simulant and stored for 10 days at 60 °C. All samples were completely covered with the simulant during storage. From each sample film containing laponite in the polymer (2%, 4%, 6% Laponite in LDPE) four equally treated samples were prepared, from the LDPE blanks three identical samples. All migration samples were injected into the AF4/MALLS system twice

Additionally, LDPE reference samples (without Laponite) and solvent blanks were prepared and stored under the same conditions.

To validate the migration experiments tests on recovery were performed. Freshly prepared laponite dispersions with 1000 ng/mL laponite were stored for 10 days at 60 °C in 2000 mg/L Novachem solution in the same vials as described above. Stored dispersions were measured by AF4/MALLS and compared to a measurement performed directly after preparation of the Laponite dispersion in a Novachem solution. The recovery rates are calculated as the ratio of the detected total peak areas (MALLS output of all detector angles except the 7° angle).

## 3. Results

### 3.1. TEM Measurements

**Figure 1:**
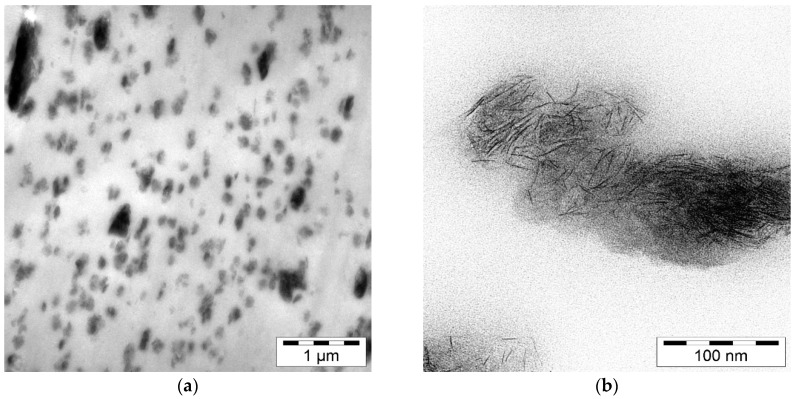
Transmission Electron Microscopy (TEM) images of the 6% laponite in LDPE film: (**a**) low and (**b**) high resolved image.

The lower resolution (Figure 1a) gives a picture of the laponite distribution in the polymer showing that the laponite exists as layered stacks which are homogenously distributed in the polymer. However, a statistical evaluation of the particle size distribution of laponite within the polymer was not performed by TEM. The high resolution image (Figure 1b) shows single laponite aggregates in the polymer. At this resolution, it can be seen that the platelets of laponite do not form compact stacks anymore but are rather oriented randomLy and not parallel to each other. At the border region of the laponite aggregates single platelets were found. This was an indication that the Laponite stacks were already intercalated and partial exfoliation of the stacks took place.

### 3.2. Characterization and Quantification of Laponite in Dispersion

**Figure 2:**
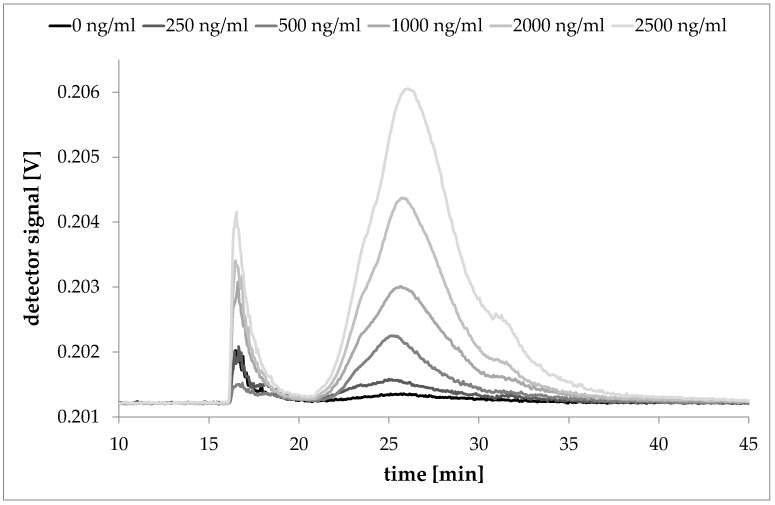
Serial dilution of laponite dispersion in 2000 mg/L Novachem solution (2000 µL injected, signal of the 92° detector).

Fractograms of laponite reference dispersions and the solvent blank are shown in Figure 2. Beside the signal caused by the Laponite particles, the 2000 mg/L Novachem dispersant blank caused a signal at elution times typical for the Laponite particles (*t* = 21 to 40 min). This is caused by a pressure drop within the AF4 channel during the fast decline of the cross flow. At an injection volume of 2000 µL each sample at a concentration between 250 ng/mL and 2500 ng/mL delivered a signal that could be distinguished from the blank and from samples with other concentrations. A 250 ng/mL laponite sample still delivered an evaluable signal. Concentrations lower than 250 ng/mL lead to problems for an explicit evaluation by light scattering. Thus, the lowest detectable concentration for laponite particles with this AF4 system and method is 250 ng/mL. At the injection volume of 2000 µL this corresponds to 500 ng of laponite.

**Figure 3:**
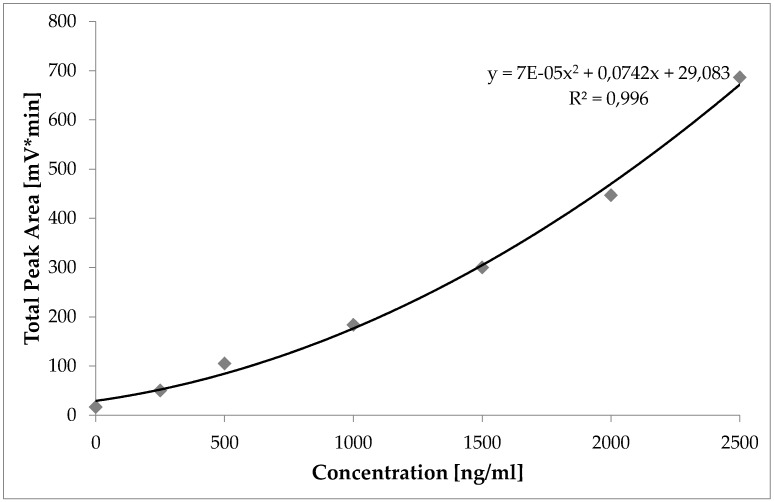
Sum of all MALLS detector angle areas (total area) of the laponite peaks versus the concentration (injection volume 2000 µL).

The signal outputs (excluding 7°, 12° and 164° detector angle) of the MALLS detector from the respective laponite reference dispersions were integrated, summed up to the total peak area and correlated with the concentration of the respective laponite reference dispersion (Figure 3). The function of this correlation experiment was used to determine laponite concentrations in unknown samples. The results of the calibration experiment are summarized in Table 1.

**Figure 4:**
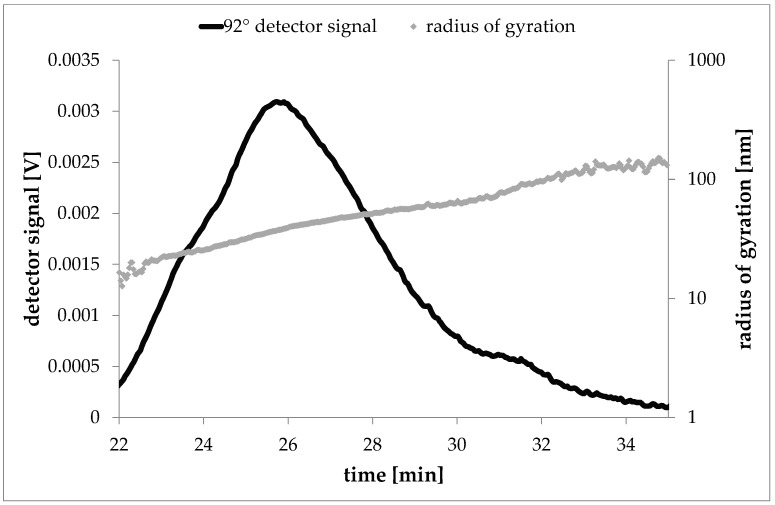
Elugram of an AF4 run with laponite particles (2000 ng/mL, 2000 µL injected. Signal of the 92° detector overlaid with the calculated radii of gyration at the relevant elution times.

Via the MALLS detection, the particle sizes of laponite particles were determined in dispersion. For the calculation of the radius of gyration a random coil fit was used. In Figure 4 the fractogram (signal intensity vs. elution time) of a 2 mg/L laponite dispersion is overlaid by the calculated radii at the respective elution times. The almost linear particle radius increase with increasing elution volume indicates a successful separation of the laponite particles. The laponite particles show a particle size distribution starting from about 16 nm to about 130 nm (radius of gyration *r_g_*). For the main part of the particles the calculated radius of gyration was about 41 nm. Radius of gyration, also root mean square radius, describes the distribution of mass around the centre of mass of the particular particle. This calculation is based on the angular variation of the signal intensities of the MALLS detector. The calculation of the radius of gyration is independent of the shape of the particle. However, *r_g_* can be re-calculated into geometrical sizes under the assumption of specific geometrical shapes [32,33]. In case of random coils, the geometrical end-to-end distance the particle size distribution ranges from 21 nm to 168 nm with the main part with 53 nm. This indicates that both, small primary units as well as smaller stacks of laponite were present in dispersion. This way, the dispersion used for AF4/MALLS method development covered the same laponite sizes as it was found in TEM images of the nanocomposites which were used in the later migration experiment.

### 3.3. Migration Test Results

**Figure 5:**
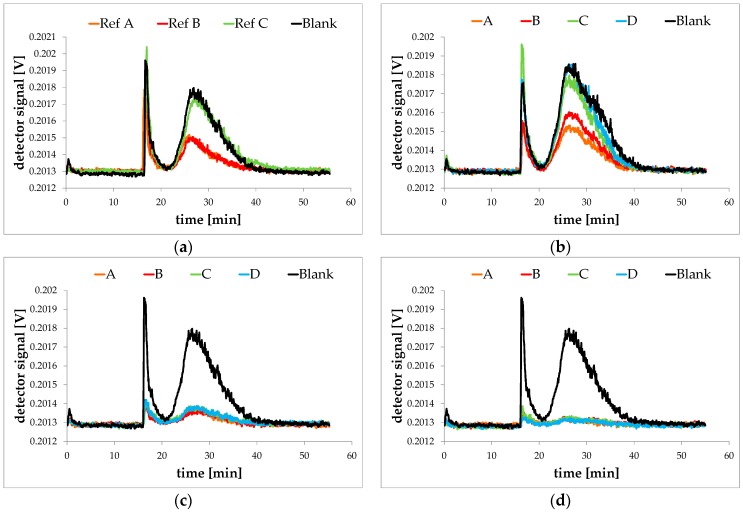
AF4/MALLS fractograms of the migration samples. (**a**) LDPE reference samples without laponite as well as nanocomposites with (**b**) 2% laponite; (**c**) 4% laponite and (**d**) 6% laponite. The black fractograms are the 2000 mg/L Novachem simulant blank that was stored for 10 d at 60 °C.

All migration experiments were carried out using a 2000-mg/L Novachem solution as simulant. Laponite particles are expected to elute from about *t* = 21 to 40 min. Due to the rapid decrease of the cross flow at that time the surfactant blank caused a slight signal (see Figure 2). AF4/MALLS measurements of the migration sample without laponite in the polymer (Figure 5a) and with 2% (Figure 5b), 4% (Figure 5c) and 6% laponite (Figure 5d) in the polymer showed, that no higher signal intensities than the surfactant blank could be detected in any sample, indicating that no oligomers were extracted and no laponite particles migrated into the food simulant after storage for 10 days at 60 °C. Rather, it appears that the surfactants of the food simulant solution were adsorbed by the polymer and especially by the laponite particles present in the polymer film or the surface of the polymer film cutting edges. This effect could especially be observed for migration samples using LDPE films with higher laponite loadings. Migration samples prepared from LDPE reference films and LDPE films with only 2% laponite in the polymer caused signals in the AF4/MALLS run exactly like or slightly lower than the surfactant blank which was also stored for 10 days at 60 °C. All migration samples prepared from the LDPE films with 4% laponite in the polymer caused signals significantly lower than the surfactant solution would do without contact to the test film. Migration samples prepared from LDPE films with 6% laponite in the polymer showed no more signal at elution times relevant for laponite particles or the surfactant solution.

**Figure 6:**
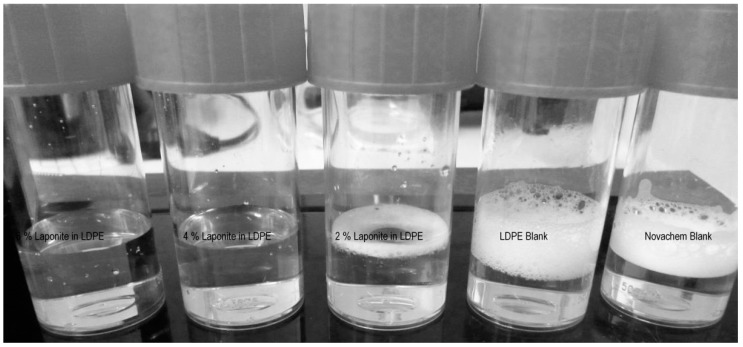
Migration samples after they were shaken. From left to right: 6% Laponite in LDPE, 4% Laponite in LDPE, 2% Laponite in LDPE, LDPE blank and the surfactant blank used as food simulant for all migration samples.

The preliminary dispersion experiments showed that the Novachem surfactant solution was suitable to disperse laponite particles. Therefore, it is conceivable that the surfactants in the simulant solution were adsorbed by the laponite particles at the surface or the cutting edges of the polymeric films. Beside the AF4/MALLS measurements, the loss of surfactant in the food simulant could be demonstrated by a simple test. Since the surfactant solution is a foam building liquid the presence of the surfactant can be visualized by shaking of the samples (Figure 6). Whilst migration samples using LDPE blanks and the pure Novachem solution were still foaming, the 2% laponite in LDPE migration samples showed already a lower ability to foam. Samples with 4% and 6% laponite in the polymer showed no foam after they were shaken.

### 3.4. Validation of Experiments

**Figure 7:**
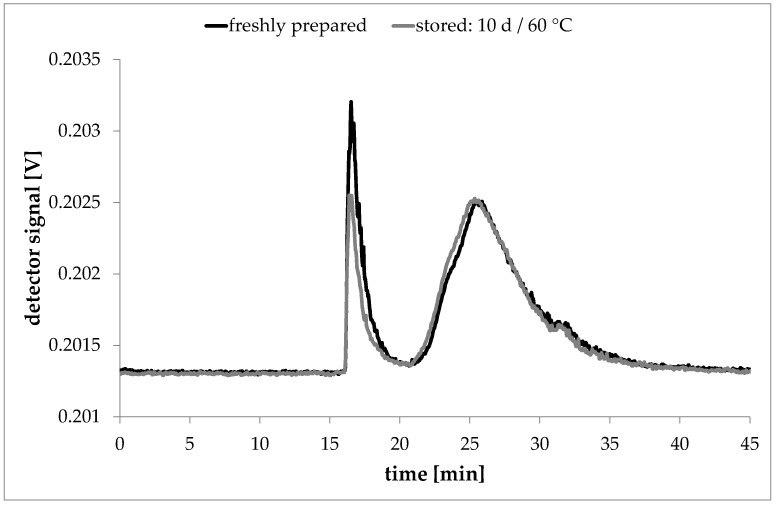
Recovery experiment for 1000 ng/mL Laponite in 2000 mg/L Novachem solution. The black curve is the fractogram of a fresh dispersion; the red curve is the fractogram of the same sample after 10 days at 60 °C (signals of the 68° detector).

To validate the migration experiments tests on recovery were performed. Freshly prepared laponite dispersions with 1000 ng/mL laponite were stored for 10 days at 60 °C in 2000 mg/L Novachem solution in the same vials as described. The dispersions in 2000 mg/L Novachem were measured by AF4 and compared to a measurement performed directly after preparation of the laponite dispersion in a Novachem solution (Figure 7). The recovery rates were calculated as the ratio of the detected total peak areas.

The storage of laponite in a Novachem solution showed that the laponite particles have good stability in the surfactant solution. The particles of the stored sample eluted at identical times as the laponite particles from the freshly prepared dispersion and caused similar signal intensities. After storage of the dispersion for 10 days at 60 °C approximately 105% of the original peak area could be recovered (see Table 2). This experiment showed that laponite would have been detectable even after storage for 10 d at 60 °C if migrated into the simulant.

**Figure 8:**
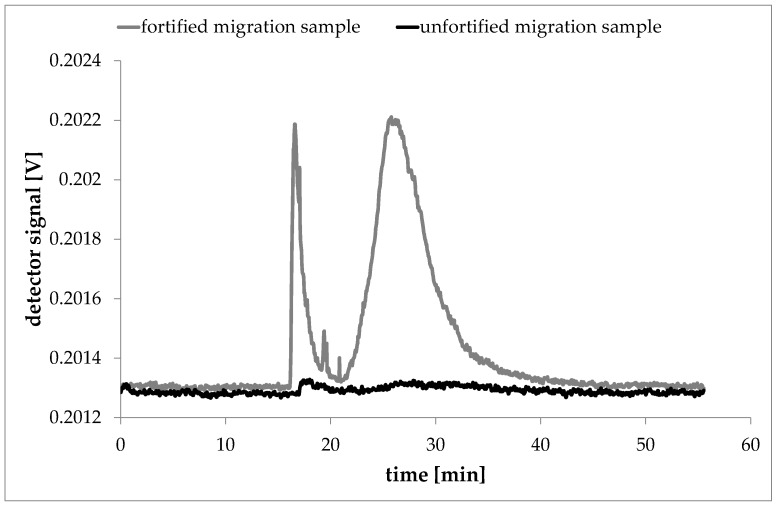
AF4 fractograms from a 6% laponite in LDPE migration solution before and after spiking to 500 ng/mL laponite.

AF4/MALLS measurements can only distinguish between samples of different sizes. For a clear differentiation between polymer, laponite particles and surfactants, the used AF4 method must be able to separate them into separated fractions. In this study, a rapid decline of the separation force (cross flow) was required, where pressure drops caused the surfactant solution to produce a slight peak in the AF4 fractogram at laponite relevant elution times. However, the presence of extracted oligomers from the polymer films or the presence of migrated laponite particles would cause higher signal intensities than dispersant blank. To further validate the method and exclude matrix effects the migration solutions were fortified with a defined amount of laponite. For the validation measurements 10 µL of a 250 µg/mL Laponite dispersion were filled up to the 5 mL mark of a volumetric flask with the respective migration sample. This way, all migration samples were fortified to a laponite content of 500 ng/mL. Injection of a migration solution made from a 6% laponite in LDPE sample was measured. The fractogram showed no signal at the retention time typical for laponite particles. After this run the fortified sample, which was prepared using the identical migration solution than before, was measured. As a result, this AF4 run shows a clear signal at elution times that were typical for the laponite particles (Figure 8).

## 4. Discussion

Due to its size with 1 nm in thickness and 25 nm in diameter only, laponite is one of the smallest clay types (in all three dimensions) and NMs (in one dimension) in general. With migration being based on size-dependent Fickian diffusion [27,34,35] lapoAnite can be considered to be a worst-case nano-additive for FCM made of plastics due to expected higher mobility than most other clay NMs. However, from a migration theory point of view, even laponite platelets are already too large to diffuse within a polymer matrix [27,36].

In this study, no detectable release of laponite particles was found, and even though migration was tested by total immersion of sample pieces where at the cutting edges direct contact of the NM with the used food simulant was possible, this migration test mode should not be applied when NMs would be of small spherical geometry because they may be more easily released from the cut edges into the immersing liquid. Interestingly, it rather appeared that instead of release of laponite particles, the surfactant of the simulant solution was adsorbed. This could happen at best via the cutting edges of the test films where direct contact of laponite with the surrounding simulant matrix was possible. This is further supported by the observed increasing effect with increasing laponite loadings in the polymer. Fortification experiments of migration solutions demonstrated that already low concentrations of laponite would have caused a signal in the AF4/MALLS fractogram. Thus, at the achieved method, detection limit migration of laponite from the nanocomposite could be excluded. From the detection limit of the device, the used amount of simulant and sample area in the migration experiments and the recovery rate under test conditions the overall detection limit of the method was 3.6 µg/dm². Assuming a surface-to-volume ratio of 6 dm² per kg food, according to the EU cube model, the filling-related detection limit was approximately 22 µg laponite per Kg food.

Other migration studies that investigated clay nanoparticles are summarized in the review of Kuorwel et al. [7]. Though there are studies that reported positive results, the possibility of artefact formation has to be considered [26]. Farhoodi et al. [37] investigated the release of organoclay from a PET-based nanocomposite. In that study, the nanocomposites were cut into several small discs and stored up to 90 d by total immersion in 3% acetic acid. The applied element specific measurements showed the presence of clay-specific elements magnesium, aluminium and silicon. Although the used method based on Inductively Coupled Plasma Optical Emission Spectrometry (ICP-OES) did not allow differentiation between particulate clay and solubilized clay elements (i.e., ionic Mg, Al and Si) the authors concluded that migration of clay was possible. This, of course, cannot be understood as a proof of particle migration. Especially in case of many cutting edges, at which direct contact of clay and the acid is possible and which was the case in that study, great care must be taken to conclude on the actually migrating species. To differentiate between particulate or ionic migrants, suitable particle-specific techniques need to be applied. 

In a study by Schmidt et al from 2011 [38] release of organically modified clay from polylactic acid (PLA) matrix was investigated. After storage of the PLA based nanocomposites in 95% ethanol the simulants were digested in acid and analysed for the presence of clay specific elements by Inductively Coupled Plasma Mass Spectrometry (ICP-MS). Likewise, to ICP-OES, normal mode ICP-MS does not allow differentiation between solubilized clay components and particulate clay. However, further particle-specific examinations with a Transmission Electron Microscope (TEM) revealed particulate structures after the acidic digestion solution was evaporated on a TEM sample holder. In such sample preparation steps artefacts might be generated when due to concentration precipitation takes place. Much more importantly, the authors measured the molecular weight (m.w.) of the PLA test samples and found severe changes of the number averaged m.w. of up to 38% during the migration test period. This means that under the aggressive migration test conditions the PLA matrix was largely destroyed by ethanolysis or hydrolysis which lead to a physical degradation of the polymer matrix followed by physical release of clay material [39]. This interpretation would be consistent with the results of a previous study by Schmidt et al. from 2009 [40]. In that study, the authors used a combination of ICP-MS and AF4/MALLS to obtain both element-specific and particle-sensitive information from the migration experiments. In 95% ethanol the authors recorded a signal with AF4/MALLS but could not find clay-specific elements with ICP-MS. The authors concluded therefore that the AF4 signals were caused by released oligomers from the PLA matrix only and not by clay particles. 

## 5. Conclusions

In this study, the potential migration of laponite particles from LDPE-based nanocomposites was investigated. In a first step, the nanomaterial was homogeneously incorporated at different NM loading concentrations into the host LDPE matrix whereby intercalation and partial exfoliation of clay stacks took place. A liquid dispersion with the same size characteristics of laponite was then prepared in an aqueous surfactant solution which was shown to give a stable dispersion of laponite under the used test conditions. This surfactant was therefore used as an appropriate food simulant for migration tests because it can be expected to disperse any migrating laponite particles. Indeed, unlike other chemical polymer additives where olive oil or 95% ethanol are the most severe food simulants, an aqueous surfactant solution can be considered as the most severe simulant in this case because of the good dispersibility and receptivity of laponite NM, once it would be released from the nanocomposite. An analytical method AF4/MALLS was successfully applied for characterization and quantification of laponite particles in the surfactant solution. As a result, no release of laponite, the smallest species of clay, was found in this study. It can therefore be concluded that diffusion-based migration of laponite particles and thus any larger structured nanoclay in general will not occur when the nanomaterial is fully embedded within a polymer matrix. In this study, a potential mechanical release of clays from the surface of the nanocomposite after severe interaction with its environment (e.g., abrasion after chemical, thermal or mechanical impacts) was not investigated. These are additional stress parameters that might appear in practice and should be considered in future research. 

## Figures and Tables

**Table 1:** Peak areas of Laponite particles obtained by Multi Angle Laser Light Scattering (MALLS) detection (2000 µL injection volume).

Concentration of Standard (ng/mL)	Mass (ng)	Total Area by MALLS (mV*min)
0	0	16.7
250	500	50.2
500	1000	105.0
1000	2000	183.7
1500	3000	300.1
2000	4000	446.9
2500	5000	686.3

**Table 2:** Recovery experiment made with a 1000 ng/mL laponite dispersion (2000 µL injections, samples prepared in triplicate).

MALLS Area “Fresh” (mV*min)	MALLS Area “Stored” (mV*min)	Recovery Rate	Direct LOD	LOD Method
182.9/184.3/183.9 183.7 (average)	192.3/197.0/189.7 193.0 (average)	105.1%	500 ng	476 ng
